# Meta-Analysis Indicates That the European GWAS-Identified Risk SNP rs1344706 within ZNF804A Is Not Associated with Schizophrenia in Han Chinese Population

**DOI:** 10.1371/journal.pone.0065780

**Published:** 2013-06-12

**Authors:** Ming Li, Hui Zhang, Xiong-jian Luo, Lei Gao, Xue-bin Qi, Pierre-Antoine Gourraud, Bing Su

**Affiliations:** 1 State Key Laboratory of Genetic Resources and Evolution, Kunming Institute of Zoology, Chinese Academy of Sciences, Kunming, Yunnan, People's Republic of China; 2 University of Rochester Flaum Eye Institute, University of Rochester, Rochester, New York, United States of America; 3 Key Laboratory of Mental Health, Institute of Psychology, Chinese Academy of Sciences, Beijing, People's Republic of China; 4 Department of Neurology, School of Medicine, University of California San Francisco, San Francisco, California, United States of America; 5 University of Chinese Academy of Sciences, Beijing, People's Republic of China; Huazhong University of Science and Technology, China

## Abstract

Recent genetic association studies have implicated several candidate susceptibility variants for schizophrenia among general populations. Rs1344706, an intronic SNP within ZNF804A, was identified as one of the most compelling candidate risk SNPs for schizophrenia in Europeans through genome-wide association studies (GWASs) and replications as well as large-scale meta-analyses. However, in Han Chinese, the results for rs1344706 are inconsistent, and whether rs1344706 is an authentic risk SNP for schizophrenia in Han Chinese is inconclusive. Here, we conducted a systematic meta-analysis of rs1344706 with schizophrenia in Chinese population by combining all available case-control samples (N = 12), including a total of 8,982 cases and 12,342 controls. The results of our meta-analysis were not able to confirm an association of rs1344706 A-allele with schizophrenia (p = 0.10, odds ratio = 1.06, 95% confidence interval = 0.99–1.13). Such absence of association was further confirmed by the non-superiority test (p = 0.0003), suggesting that rs1344706 is not a risk SNP for schizophrenia in Han Chinese. Detailed examinations of individual samples revealed potential sampling bias in previous replication studies in Han Chinese. The absence of rs1344706 association in Han Chinese suggest a potential genetic heterogeneity in the susceptibility of schizophrenia on this locus and also demonstrate the difficulties in replicating genome-wide association findings of schizophrenia across different ethnic populations.

## Introduction

Schizophrenia is one of the most severe complex psychiatric disorders, and a lifetime prevalence of schizophrenia is estimated at 0.70% to 1.10% in general populations worldwide [1]. There is a strong genetic component to these risks–the heritability of schizophrenia has been estimated to be about 80% [2] and the risk of siblings of an affected individual is about eightfold to tenfold greater than general populations [3], [4]. These findings imply that the application of genetic analyses to schizophrenia seems plausible and timely.

Schizophrenia is widely acknowledged to be a polygenic disorder, involving both common variants with low effect size and rare variants with high effect size [5], and over the past few decades efforts in detecting common variants of genetic risks for schizophrenia have increased. Recent genome-wide association studies (GWASs) on schizophrenia in populations of European ancestry have implicated many risk common genetic variants with small to moderate effect size, including rs1625579 in Chr.1p21.3 region (p = 1.59×10^−11^, odds ratio = 1.12) [6], rs6932590 in Chr.6p22.1 region (p = 1.4×10^−12^, odds ratio = 1.16) [7], and rs11819869 in Chr.11p11.2 region (p = 3.89×10^−9^, odds ratio = 1.25) [8]. Among these risk variants achieving genome-wide level of statistical significance, rs1344706, a SNP located in intron 4 of ZNF804A (Chr.2q32.1), is one of the most compelling polymorphisms.

Rs1344706 was firstly identified by a GWAS of a sample from the UK, in combination with a follow-up replication study among different world populations [9], though it did not achieve the conventional genome-wide significance level. Subsequent replication studies in Europeans by independent study groups have, however, demonstrated that rs1344706 is likely an authentic risk SNP for schizophrenia in Europeans [10]–[12]. More importantly, a recent meta-analysis of a large data set (18,945 cases and 38,675 controls) found genome-wide significant association of rs1344706 with schizophrenia (p = 2.5×10^−11^), and the result was more strengthened when bipolar disorder samples were added into the meta-analysis (p = 4.1×10^−13^) although the effect size is relatively small (odds ratio = 1.10, 95% confidence interval = 1.07–1.14) [13].

The significant association of rs1344706 was firstly replicated in a small Han Chinese sample (566 cases/574 controls from Xi’an, China) [14], suggesting it may also be a risk SNP for schizophrenia among Chinese. However, other studies on rs1344706 using different Han Chinese samples yielded inconsistent results. Xiao et al. [15] and Chen et al. [16] observed the association in two other Han Chinese samples (from Xinxiang and Shandong, China), but negative results have also been frequently reported. O’Donovan et al. [9] found that rs1344706 was not significant in the Han Chinese sample from Shanghai, similar to a study of Han Chinese samples from Sichuan reported by Steinberg et al. [12]. Recently, we showed that rs1344706 was not associated with schizophrenia in two Han Chinese samples from southwestern China (Yuxi and Kunming, China) [17], similar to another Chinese sample from Singapore [18]. Most recently, a large-scale GWAS (3,750 cases/6,468 controls) in Han Chinese found no association for rs1344706 in their samples recruited from the northern, central, and southern parts of China, p = 0.71) [19]. Overall, whether rs1344706 confers genetic risk of schizophrenia in Han Chinese or not is still inconclusive.

To test whether rs1344706 is a real risk SNP for schizophrenia in Chinese population, in the present study we conducted a comprehensive meta-analysis by combining all available Han Chinese case-control samples (8,982 cases and 12,342 controls).

## Results

### Literature Search and Eligible Studies

**Figure 1:**
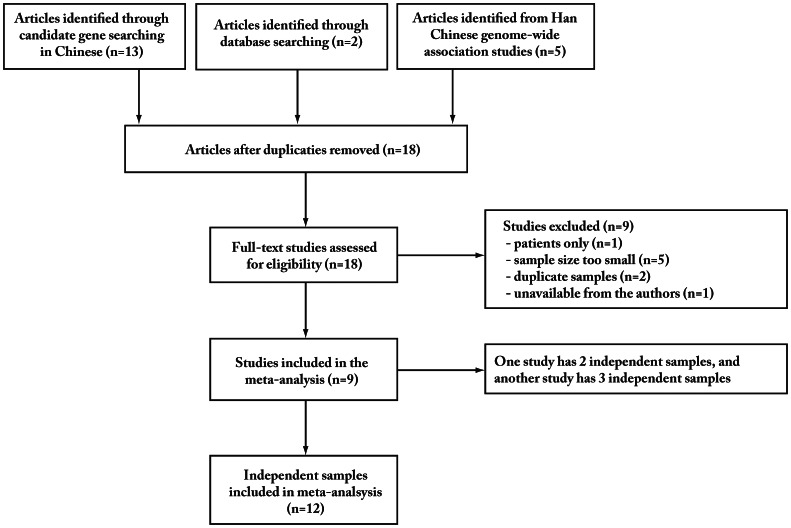
Literature search flow chart.

Using our literature search approaches (a flow chart of the search process is detailed in **Figure 1** **)**, a total of nine studies in Chinese population were identified and included in the meta-analysis [9], [12], [14]–[20]. Briefly, there are four replication studies for rs1344706 in Chinese having one or two samples [14]–[17] that were included in our meta-analysis. Additionally, there are three previous meta-analyses of rs1344706 with schizophrenia in world populations [9], [12], [18] having Chinese samples independently from the four replication studies and we extracted the Chinese samples from these for meta-analysis: a Shanghai sample from O’Donovan et al. [9], a Sichuan sample from Steinberg et al. [12], and a Singapore sample from Li et al. [18]. Furthermore, two independent Han Chinese GWASs were also included [19], [20].

Among these nine studies, a Han Chinese GWAS [19] has three independent samples and a replication study [17] has two independent samples. Therefore, a total of nine studies with twelve independent samples were included in our meta-analysis. The participants in each sample were recruited from local volunteers and there was no overlap among these studied samples. **Table 1** lists the information of the twelve samples, including mean age, gender ratio, criteria on definition of schizophrenia, sample size, etc.

**Table 1:** Characteristics of included studies (and samples) on the association of rs1344706 with schizophrenia in Han Chinese.

Author, year	Sample area	Schizophrenia Cases			Healthy Controls			Definition of schizophrenia	Genotyping method
		N^a^	Mean Age	Gender (% Male)	N^a^	Mean Age	Gender (% Male)		
Chen, 2012 [16]	Shandong	570	28.2±7.8	61.5	448	23.0±7.0	65.1	ICD-10	TaqMan
Li, 2011 [17]	Yuxi	488	38.5±10.4	53.1	694	37.1±6.8	53.5	ICD-10	SNaPShot
Li, 2011 [17]	Kunming	403	36.3±8.7	44.4	604	36.6±7.0	44.4	DSM-IV	SNaPShot
Li, 2012 [18]	Singapore	885	49.0±13.2	66.1	976	46.1±10.6	63.3	DSM-IV	Illumina 1M
Liou, 2012 [20]	Taiwan	522	44.1±9.1	55.4	793	67.4±9.4	57.5	DSM-IV	Affymetrix 6.0
O’Donovan, 2008 [9]	Shanghai	996	38.8±14.1	55.1	1,015	30.0±8.7	50.5	DSM-IV	TaqMan
Shi, 2011 [19]	Shanghai and Anhui	1,224	36.2±12.4	55.9	2,788	60.9±12.2	35.5	DSM-IV	Affymetrix 6.0
Shi, 2011 [19]	Beijing and Shandong	1,510	36.9±9.3	69.8	1,546	30.8±11.1	50.3	DSM-IV	Affymetrix 6.0
Shi, 2011 [19]	Guangdong and Guangxi	883	36.3±16.6	58.4	2,010	56.1±13.5	47.7	DSM-IV	Affymetrix 6.0
Steinberg, 2010 [12]	Sichuan	439	NA	53.5	446	NA	50.0	DSM-IV	NA
Xiao, 2011 [15]	Xinxiang	496	28.6±7.6	53.0	448	29.2±7.9	47.6	DSM-IV	RFLP
Zhang, 2010 [14]	Xi’an	566	*see below	52.1	574	*see below	57.3	DSM-IV	TaqMan

NA, not available from the published study;

DSM-IV, diagnosis and statistical manual of mental health disorders, fourth edition;

ICD-10, the international classification of diseases 10;

* In the Xi’an sample, the mean age of cases and controls were calculated in males and females separately. In cases, males mean age  = 35.2±11.9, and females mean age = 32.6±13.7; in healthy control subjects, males mean age  = 29.0±14.1, females mean age  = 29.3±13.5;

a The N represents the number of individuals having data for rs1344706.

We should note that while some other studies discuss the connection between ZNF804A and schizophrenia in Han Chinese, they were not included in our meta-analysis either due to small sample size, being a case-only study, using samples that overlap with those already included in our meta-analysis, or where data was not available from the authors of these studies. The list of these studies is shown in **Table S1**.

Among these twelve independent case-control samples, three of them reported significant association of rs1344706 with schizophrenia in Chinese [14]–[16], while the other nine did not 9,12,17–20. To test if rs1344706 is an authentic risk SNP for schizophrenia in Chinese, we performed a series of analyses, and discussed the reasons for this inconsistency.

### Assessment of Publication Bias

**Figure 2:**
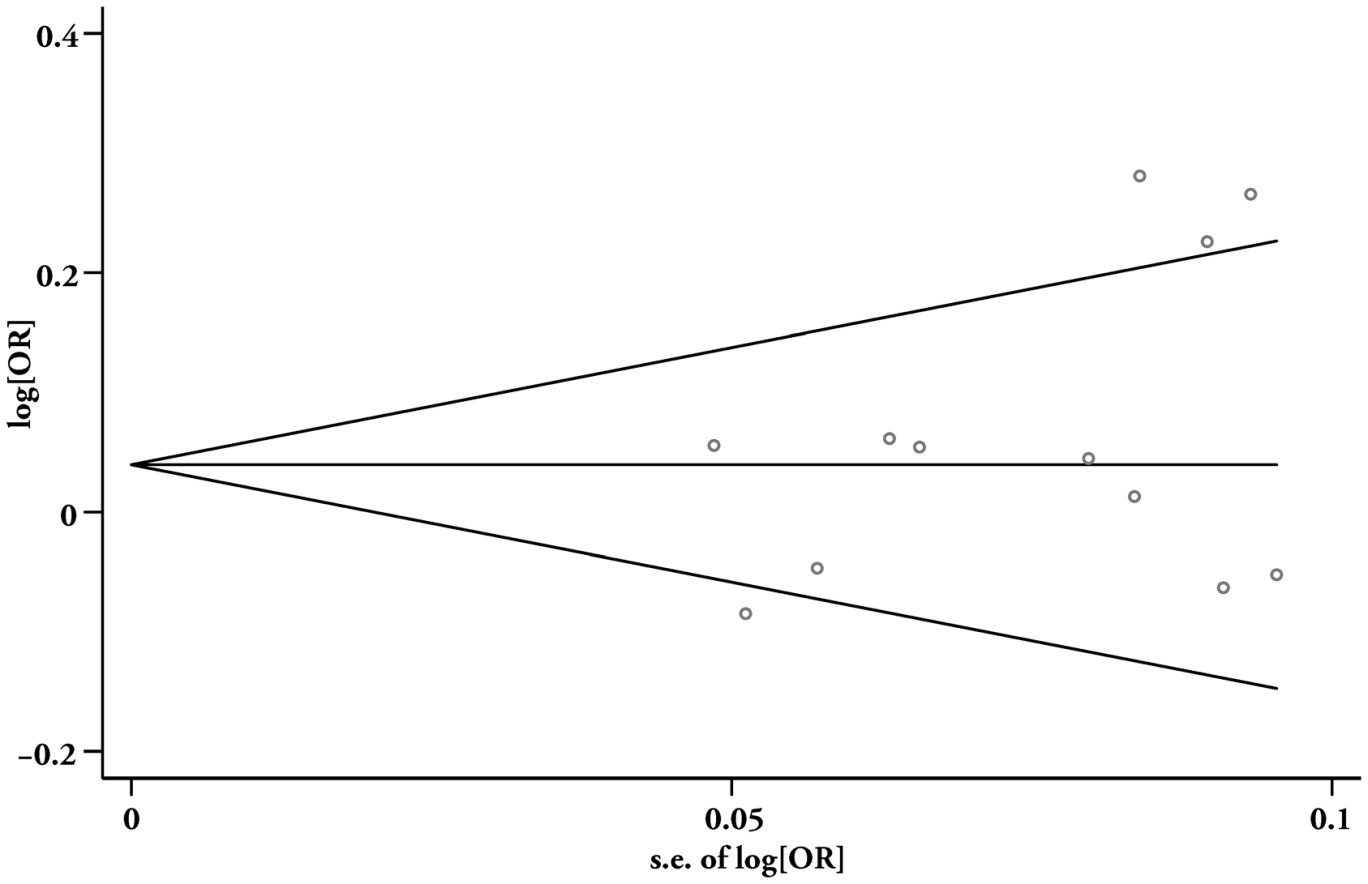
Begg’s funnel plot with pseudo 95% confidence limits for meta-analysis of rs1344706.

STATA 11.0 software (Stata Corp LP, College Station, Texas, United States) was used to detect the presence of potential publication bias. Begg’s funnel plot (**Figure 2**) for meta-analysis of rs1344706 seems symmetrical (Begg’s p  = 0.63), suggesting no evidence of publication bias. Egger’s regression test also showed no evidence of publication bias (p = 0.14, 95%CI = [−1.16, 7.07]).

### Regression Analyses on Potential Influential Factors

We extracted the data for mean age and gender ratio from each study (**Table 1**), and then calculated the allelic-specific odds ratios for rs1344706 using allele counts in cases and controls for each study (**Table 2**). Afterward, we performed regression analyses to test if these two factors could influence the odds ratios. Since data of mean age was not available in the study by Steinberg et al. [12], their sample from Sichuan was not included in this analysis. Regression analyses showed that the odds ratios for rs1344706 were not influenced by mean age in patients with schizophrenia (p = 0.16) or in healthy controls (p = 0.12), and the odds ratios were also not influenced by the gender ratio among schizophrenia patients (p = 0.48) or healthy subjects (p = 0.35).

**Table 2:** Meta-analysis of rs1344706 with schizophrenia in Han Chinese samples.

Author, year	Sample area	N Cases/N Controls	A-allele frequencies		P-value	OR (95%CI)	
			Cases	Controls			
Chen, 2012 [16]	Shandong	570/448	0.564	0.508	0.013	1.25 (1.05–1.49)	
Li, 2011 [17]	Yuxi	488/694	0.506	0.503	0.876	1.01 (0.86–1.19)	
Li, 2011 [17]	Kunming	403/604	0.500	0.516	0.489	0.94 (0.79–1.12)	
Li, 2012 [18]	Singapore	885/976	0.520	0.506	0.389	1.06 (0.93–1.20)	
Liou, 2012 [20]	Taiwan	522/793	0.512	0.501	0.57	1.05 (0.89–1.22)	
O’Donovan, 2008 [9]	Shanghai	996/1,015	0.530	0.514	0.166	1.06 (0.94–1.20)	
Shi, 2011 [19]	Shanghai and Anhui	1,224/2,788	0.517	0.503	0.25	1.06 (0.96–1.16)	
Shi, 2011 [19]	Beijing and Shandong	1,510/1,546	0.480	0.501	0.10	0.92 (0.83–1.02)	
Shi, 2011 [19]	Guangdong and Guangxi	883/2,010	0.494	0.506	0.42	0.95 (0.85–1.07)	
Steinberg, 2010 [12]	Sichuan	439/446	0.533	0.546	0.62	0.95 (0.79–1.15)	
Xiao, 2011 [15]	Xinxiang	496/448	0.600	0.536	0.005	1.30 (1.09–1.57)	
Zhang, 2010 [14]	Xi’an	566/574	0.527	0.457	0.00083	1.32 (1.12–1.56)	
**All Chinese samples**		8,982/12,342	0.518	0.506	0.10	1.06 (0.99–1.13)	

OR, odds ratio; CI, confidence interval.

Test of heterogeneity: χ^2^ = 29.23, df = 11, p = 0.002.

*I* ^2^ (variation in OR attributable to heterogeneity) = 62.4%.

The result for the combined samples (p = 0.10, Z = 1.62) was assessed using the Mantel-Haenszel method with the random-effects model.

The frequency of A-allele in the Xi’an control sample is highlighted in gray.

To further test if the inconsistent association of rs1344706 in Chinese was caused by other factors, e.g. geography, we first ascertained the sample collection area of each study according to information provided in their published papers and categorized these area into Northern, Central and Southern China, and conducted regression analysis with the odds ratios. The result was not significant (p = 0.47). Furthermore, we obtained the latitude of each sample collection area and tested their associations with the odds ratios for rs1344706, and still no significant associations were observed (p = 0.40).

### Power Analysis

Before performing the meta-analysis, we conducted a power analysis (**Figure S1–A**) on our total sample size using the following assumptions: 8,982 patients with schizophrenia and 12,342 controls, two-tailed α = 0.05, and the frequency of A-allele in Chinese (0.5206). Given the inconsistent odds ratios of rs1344706 for schizophrenia in Han Chinese, we used two commonly observed odds ratios in genetic association studies, 1.10 and 1.20. The present sample size revealed a >92% power of detecting a significant association of allele given an odds ratio of 1.10 (corresponding to a “weak” gene effect). When an odds ratio of 1.20 was assumed, i.e. a “weak to moderate” gene effect, our sample size showed >99% power to detect significance (α<0.05).

### Meta-analysis of rs1344706 with Schizophrenia

**Figure 3:**
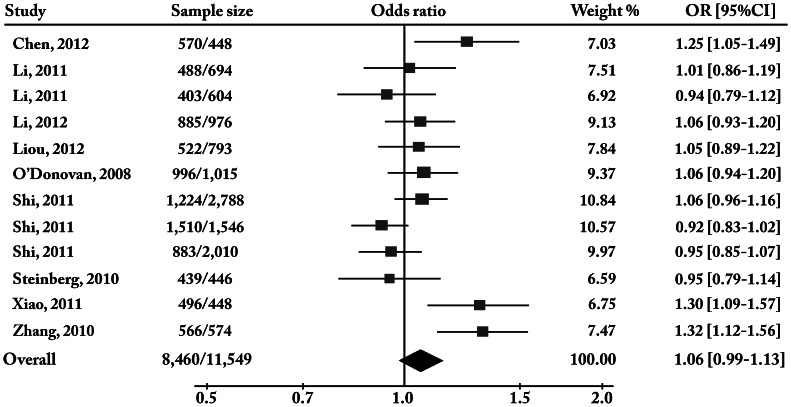
Forest plot of meta-analysis for rs1344706 [A] in Chinese sample. “OR” is the odds ratio for each individual sample. “Overall” refers to the combined sample.

Overall, we recruited a total sample size of 21,324 subjects for meta-analysis, including 8,982 cases and 12,342 controls. The analysis of the allelic association of the A-allele with the risk of schizophrenia revealed significant heterogeneity among the twelve individual samples (χ^2^ = 29.23, df = 11, p = 0.002), and meta-analysis (Z-score test) of the combined samples was assessed with the random-effect model. By combining the twelve samples in the meta-analysis, we found that rs1344706 was not associated with schizophrenia (Z = 1.62, p = 0.10, odds ratio = 1.06, **Table 2**). The forest plot of the meta-analysis is presented in **Figure 3**.

A detailed examination of the association results of rs1344706 in Han Chinese samples indicated that the Xi’an sample [14] showed the lowest p-value (p = 0.00083), but the frequency of A-allele in their control sample is 0.457 (highlighted in **Table 2**), highly different from studies in other Chinese control samples (shown in **Table 2**) as well as the Han Chinese data from the 1000-Human-Genome (the “A” allele frequency is 0.5206). This striking difference can hardly be explained by currently known genetic divergence between southern and northern Chinese populations [21], because no significant difference was observed among the other Chinese samples, suggesting the possibility of a biased sampling of the control sample in the Xi’an study [14] that subsequently led to a false positive result. Hence, whether the data from the Xi’an sample should be considered as an authentic result for rs1344706 is questionable.

Although Xiao et al. [15] and Chen et al. [16] also reported significant associations of rs1344706 with schizophrenia, their samples were much smaller as compared with the other Chinese samples, and their results may not be very representative.

### Sensitivity Analyses

‘Leave-one-out’ sensitivity analysis to determine whether the meta-analysis results were driven by any sample data and therefore suggestive of a ‘winner’s curse’ phenomenon [22] revealed that removal of any of the included samples still led to the non-significant (or marginal significant) association of rs1344706 with schizophrenia using the eleven remaining samples (**Table S2**). Furthermore, we noticed that when the Xi’an sample was removed from the meta-analysis, the estimates of the p-value for the heterogeneity test increased from 0.002 to 0.03, suggesting that the genetic heterogeneity was mainly caused by the Xi’an sample, and when the Xi’an sample was removed from the meta-analysis, the p-value remained non-significant (Z = 1.13, p = 0.26, odds ratio = 1.03, **Table S2**).

### Equivalence-based Analysis to Test the Absence of Association between rs1344706 and Risk of Schizophrenia

Non-superiority test was conducted to confirm the absence of association between rs1344706 and schizophrenia in Chinese. The null hypothesis is that the frequency of A-allele at rs1344706 in schizophrenia patients is greater by 3% than that in controls. The 3% was set based on the reported differences of rs1344706 A-allele between schizophrenia patients and healthy controls in the discovery UK GWAS [9] and the first European replication study [10] as well as the first Chinese replication study [14], i.e. 7.0%, 4.0% and 7.0% respectively. Thus, a 3% excess in cases can be regarded as a the lower bound of previous estimations, which would correspond to an odds ratio of 1.12 according to the allele distribution of rs1344706 in Han Chinese.

The non-superiority p-values are presented **Table 3**. Overall, the result of the combined Chinese case-control samples supports the absence of association (p = 0.0003), suggesting that the excess of rs1344706 A-allele in the cases is lower than 3% (i.e. odds ratio<1.12), which support the absence of association between rs1344706 and schizophrenia in Han Chinese.

**Table 3:** Non-superiority tests of rs1344706 with schizophrenia in Han Chinese samples.

Author, year	Sample area	N Cases/N Controls	A-allele frequencies		Delta of A-allele carrier frequency (%)	Nonsuperiority P-value H0: A frequency, cases>controls +3%
			Cases	Controls		
Chen, 2012 [16]	Shandong	570/448	0.564	0.508	5.6	0.8939
Li, 2011 [17]	Yuxi	488/694	0.506	0.503	0.3	0.1264
Li, 2011 [17]	Kunming	403/604	0.500	0.516	−1.6	0.0320
Li, 2012 [18]	Singapore	885/976	0.520	0.506	1.4	0.1751
Liou, 2012 [20]	Taiwan	522/793	0.512	0.501	1.1	0.1978
O’Donovan, 2008 [9]	Shanghai	996/1,015	0.530	0.514	1.6	0.1983
Shi, 2011 [19]	Shanghai and Anhui	1,224/2,788	0.517	0.503	1.4	0.1396
Shi, 2011 [19]	Beijing and Shandong	1,510/1,546	0.480	0.501	−2.1	0.0000
Shi, 2011 [19]	Guangdong and Guangxi	883/2,010	0.494	0.506	−1.2	0.0066
Steinberg, 2010 [12]	Sichuan	439/446	0.533	0.546	−1.3	0.0384
Xiao, 2011 [15]	Xinxiang	496/448	0.600	0.536	6.4	0.9237
Zhang, 2010 [14]	Xi’an	566/574	0.527	0.457	7.0	0.9918
All Chinese samples		8,982/12,342	0.518	0.506	1.2	0.0003

For each of the samples, a non**-**superiority p-value is reported that corresponds to the statistical significance of the null hypothesis that the frequency of the rs1344706 A-allele is greater in schizophrenia cases than in controls and differs by at least 3%.

## Discussion

Recently, O’Donovan et al. undertook a GWAS with 479 UK schizophrenia cases and 2,937 controls in tandem with a follow-up replication study of 16,726 additional subjects, and found that rs1344706 in ZNF804A is significantly associated with schizophrenia [9]. Subsequently, the association of rs1344706 with schizophrenia was consistently reported by the Irish Case/Control study of schizophrenia (ICCSS) [10], the International Schizophrenia Consortium (ISC) [23], the Molecular Genetics of Schizophrenia (MGS) [24] and then in a large-scale meta-analysis of independent samples including mainly European subjects [12]. A further meta-analysis of rs1344706 in 18,945 schizophrenia patients and 38,675 controls supported this association between rs1344706 and schizophrenia (p = 2.5×10^−11^), though again, the sample mostly consisted of European subjects [13]. These data strongly indicated that rs1344706 is a promising risk SNP for schizophrenia in European populations, but as we pointed out earlier the association is less clear among other populations, notably Han Chinese.

We conducted a meta-analysis combining all available Han Chinese case-control samples to test this association in a non-European population and found that rs1344706 was not associated with schizophrenia in the combined samples, and the effect size of rs1344706 for schizophrenia was smaller in Chinese than in Europeans (odds ratio for A-allele, 1.06 in Chinese vs. 1.12 in Europeans) [9]. This was not surprising, considering that rs1344706 is a genome-wide significant risk SNP for schizophrenia in Europeans [9], [13]. We then compared our effect size with the independent replication studies in Europeans, and found that the effect size in our analysis was still smaller than the results reported by the ICCSS (odds ratio = 1.20, p = 0.0113) [10], the ISC (odds ratio = 1.08, p = 0.029) [23], and the MGS samples (odds ratio = 1.09, p = 0.0262) [24]. The differences of rs1344706 in association with schizophrenia between Europeans and Han Chinese likely then reflects the genetic heterogeneity often observed in the genetic association analyses for complex diseases, probably as a result of differential population histories. Other population specific factors, such as diet, culture, or environmental exposure may also contribute to this observed heterogeneity.

We further compared the sample size between our meta-analysis (case/control, 8,982/12,342) and the studies in Europeans, and found that our total sample size was larger than the discovery GWA+replication studies on rs1344706 in O’Donovan et al. (7,308/12,834, p = 1.61×10^−7^) [9], but smaller than the meta-analysis in Steinberg et al. (5,077/20,506, p = 0.0029) [12]. The power of our sample, however, was larger (97.6%) than those two samples (96.1% and 93.5%, respectively) under the same assumption on the reported OR of 1.12 in Europeans [9] and the risk allele (A) frequency of 0.6235 in healthy European populations (**Figure S1–B**). In addition, our sample size was much larger than the samples from ICCSS (1,021/626) [10], ISC (2,519/2,110) [23] and MGS (3,967/3,624) [24] (**Figure S1–C**), in which all three found significant associations for rs1344706 whereas we did not. These comparisons further suggest that the non-significant association for rs1344706 in our meta-analysis was not caused by the sample size, but is likely due to the relatively small effect size of this SNP in Han Chinese.

We have demonstrated that our sample has enough power to detect significance using the odds ratios corresponding to small effect size (odds ratio = 1.10) and small-to-moderate effect size (odds ratio = 1.20). However, if we use the observed odds ratio (1.06) in Chinese, the present sample size only showed 55.4% power of detecting significant association. Alternatively, given the observed odds ratio (1.06) is authentic, the sample size would have to be increased to more than 24,786 cases and 24,786 controls in order for the sample to have a >90% power of detecting a significant association (a<0.05). Based on these assumptions, studies on rs1344706 in small Chinese case-control samples are unlikely to observe significant results. That said, there are three previous studies reporting significant associations of rs1344706 in small samples [14]–[16] with large effect sizes (1.25≤odds ratio≤1.32), almost achieving the effect size of genome-wide scan in UK samples (odds ratio = 1.38). This is inconceivable given the notion of the winner’s curse, that we would expect a much smaller effect size in replication studies as compared with the discovery GWASs. Accordingly it is difficult to judge whether the reported positive results were real or caused by chance.

In the meta-analysis of the twelve Chinese case-control samples, it should be noted that we did not perform population stratification analysis in the combined samples. However, among these twelve individual samples, there is no obvious population stratification in the Shanghai [25], Taiwan [20], Yuxi and Kunming [17] samples which were reported in previous studies. There is also no population stratification in Singapore sample (λ = 1.012) calculated using genome-wide SNPs (Illumina 1M array). Additionally, the Shanghai and Anhui, Beijing and Shandong, and Guangdong and Guangxi samples were merged as the primary GWAS samples in the GWAS of Shi et al. [19], and there was no population stratification in these samples. The four remaining samples from Sichuan, Xi’an, Xinxiang, and Shandong have not been tested for population stratification, and notably, three of these show significant association for rs1344706 (Xi’an, Xinxiang and Shandong) [14]–[16]. Consequently, we cannot exclude the possibility that the positive results in these three samples were caused by potential population stratification.

The data presented in our analysis is limited, and consequently we are cautious in the interpretation of our results or making any definitive conclusions. In this study, we only tested rs1344706, and the other SNPs in ZNF804A were not studied in most of the analyzed Chinese samples. However, in a previous study, we performed a relatively systematic analysis of the ZNF804A region (up to 111 SNPs) in several Han Chinese samples (Singapore, Yuxi, Shanghai and Anhui, Beijing and Shandong, and Guangdong and Guangxi), which we did include in the present study and found that most of the common SNPs in ZNF804A were not associated with schizophrenia in Chinese populations [18].

Through our meta-analysis of all the available Han Chinese case-control samples, we did not find evidence for association of rs1344706 with schizophrenia. This is not unexpected, given that the effect size of rs1344706 to schizophrenia risk in Chinese is smaller than that found in Europeans. Moreover, because a non-significant difference test cannot be interpreted as acceptance of the null hypothesis, the equivalence-based method that provides the possibility of observing a lack of association by chance was conducted to avoid the false-negative results in this study. The p-value of the non-superiority test for rs1344706 is 0.0003 in the combined Chinese samples, supporting the absence of association between rs1344706 and schizophrenia. Interestingly, rs1344706 is not the only SNP showing large differences in associations with schizophrenia between Han Chinese and Europeans, and similar situations were also observed for some other GWAS-identified SNPs in Europeans including NRGN rs12807809, RELN rs7341475, and CNNM2 rs7914558 [6], [7], [26], all of which were reported to be not significant in Chinese [25], [27], [28].

Collectively, the current data to date does not support speculations that rs1344706 is a risk SNP for schizophrenia in Chinese, and the failures of replicating rs1344706 in the present sample suggest a potential genetic heterogeneity of schizophrenia susceptibility on this locus, likely resulting from large differences in linkage disequilibrium patterns of this genomic region covering rs1344706 between European and Han Chinese, as demonstrated in our previous study [18]. Moreover, for schizophrenia and other mental disorders, there is marked phenotypic heterogeneity of clinical symptoms [29], which could confound genetic association results between different ethnic populations. Consequently, the present study invites to be careful in inferring replication results of complex psychiatric disorders, such as schizophrenia, across different ethnic populations.

## Materials and Methods

### Systematic Literature Search

The research protocol was approved by the internal review board of Kunming Institute of Zoology, Chinese Academy of Sciences. We firstly considered all the studies listed for ZNF804A on the SZGene database [30] and also searched PubMed with the search terms ‘ZNF804A’ and ‘schizophrenia’ as well as ‘schizophrenia’ and ‘GWAS’. Once the articles had been collected, their bibliographies were then searched for additional references. Studies published before January 19, 2013 were considered in this analysis. We read all the relevant papers to see if the studies used Chinese samples. After this preliminary literature search, eighteen non-duplicate studies were identified, including thirteen candidate gene studies using Chinese samples and five GWASs in Han Chinese. Whether they were then to be included in the meta-analysis was determined according to further inclusion criteria as the following section describes.

### Selection of Studies for Inclusion

Eligible studies in the meta-analysis must meet the following criteria: (1) be case-control studies, case-only and family-based studies were excluded; (2) contain at least 100 cases and 100 controls; (3) case status being defined as having diagnosis of schizophrenia according to the DSM-IV or ICD-10 criterion assessed by established psychiatric interviews, with control subjects having no history of mental disorder, other neurological disorder, alcohol dependence, or drug dependence; (4) studies where the samples have no overlap with the other identified studies; (5) rs1344706 was genotyped and in Hardy-Weinberg equilibrium in healthy controls (p>0.05).

### Data Extraction

For each candidate gene study, the following data was extracted: (1) author(s) and year of publication; (2) methods, including study design, sample size, sample collection area, definition of case status, and genotyping method; (3) sample characteristics, i.e., gender ratio and mean age; and (4) data for rs1344706 (allele counts). All the required data were available in the published studies or from supplementary information, except for one study that contained no information on the mean age and genotyping method [12].

For the GWASs, we recruited the following data: (1) author(s) and year of publication; (2) methods; and (3) sample characteristics. However, if rs1344706 was not available from the main text or supplementary information of these GWASs, we contacted the corresponding authors to request access to the data. The authors of two GWASs provided the data for rs1344706 [19], [20], but the author of another GWAS did not reply to our requests [31], and was accordingly excluded from further analysis.

Data from each study was extracted independently by two investigators (ML and HZ), using a standardized data extraction form. In cases of disagreement of study inclusion, a third investigator was involved (XJL). Disagreement over eligibility of a study was resolved by discussion until a consensus was reached.

### Statistical Analysis

Publication bias was assessed visually using a funnel plot and tested with Egger’s regression test [32] as well as the Begg’s test, which is based on Kendall’s-τ [33], with p<0.10 being considered statistically significant.

A random effects regression of odds ratio with mean age and the proportion of males as covariates was performed with the use of SPSS 16.0 (SPSS inc, Chicago, IL, USA) to determine whether these covariates could influence odds ratios for rs1344706. We also analyzed whether the sample collection area could impact the observation of odds ratios.

Power analysis was performed by the Power and Sample Size Program software [34], and the commonly observed odds ratio of 1.10 and 1.20 were applied in the power analysis, which correspond to a “weak” gene effect and a “weak to moderate” gene effect, respectively.

Meta-analysis examined the allelic association of the rs1344706 A-allele with the risk of schizophrenia relative to the C-allele (odds ratio for A-allele). Allele frequencies and counts were extracted for cases and controls. For some studies, allele frequencies and counts were calculated from the available data [12].

To combine the individual studies, we conducted meta-analyses using Review Manager 4.2.2 (http://ims.cochrane.org/revman/download/revman-4). The heterogeneity between individual studies was tested using the Cochran’s (Q) χ^2^ test, which is a weighted sum of the squares of the deviations of individual odds ratio estimates from the overall estimate. When the odds ratios are homogeneous, Q follows a χ^2^ distribution with degrees of freedom. If P_Q_ <0.10, the heterogeneity is considered statistically significant. Inconsistency across studies was quantified with the *I* ^2^ metric (*I* ^2^ = Q-d.f./Q), which can be interpreted as the percentage of total variation across several studies due to heterogeneity. *I* ^2^ takes values between 0 and 100%, with higher values denoting a greater degree of heterogeneity (0–25%: no heterogeneity; 25–50%: moderate heterogeneity; 50–75%: large heterogeneity and 75–100%: extreme heterogeneity). In the presence of heterogeneity among individual studies, we used random-effects models to combine the sample and to calculate the odds ratio and the corresponding 95% confidence interval (CI); otherwise, a fixed-effect mode was used. We used a forest plot to graphically present the pooled odds ratios and the 95% CIs. Each study was represented by a square in the plot, and the weight of each study was also shown. P<0.05 was considered statistically significant.

Sensitivity analysis was conducted to assess the potential influences of any one single study on the pooled odds ratios. Within each meta-analysis, included studies were removed one at a time to check for significant alterations to pooled odds ratios and associated p-values.

The non**-**superiority test was conducted to confirm the absence of association between rs1344706 and schizophrenia, and this equivalence-based analysis were performed with STATA 11.0 (Stata Corp LP, College Station, Texas, United States), using the command ‘equip’. Detailed descriptions of the nonsuperiority test can be found in previous studies [35], [36].

## Supporting Information

Checklist S1 **PRISMA Checklist.**
(DOC)

Flow Chart S1 **PRISMA Flow Chart.**
(DOC)

Figure S1 **Power analysis for the studied samples.**
(DOC)

Table S1 **Studies referring ZNF804A and schizophrenia in Han Chinese, but not meet the inclusion criteria for the meta-analysis.**
(DOC)

Table S2 **‘Leave-one-out’ sensitivity analysis for meta-analysis.**
(DOC)
