# Supplementary material for: Meta-Analysis Indicates That the European GWAS-Identified Risk SNP rs1344706 within ZNF804A Is Not Associated with Schizophrenia in Han Chinese Population
Source: PLoS One. 2013 Jun 12;8(6):e65780. doi: 10.1371/journal.pone.0065780 (PMC3680487; doi:10.1371/journal.pone.0065780)
Supplement: Checklist S1 — PRISMA Checklist. (DOC) [file pone.0065780.s001.doc]

| **Section/topic** | **#** | **Checklist item** | **Reported on page #** |
| --- | --- | --- | --- |
| **TITLE** | | |  |
| Title | 1 | Meta-analysis indicates that the European GWAS-identified risk SNP rs1344706 within ZNF804A is not associated with schizophrenia in Han Chinese population | 1 |
| **ABSTRACT** | | |  |
| Structured summary | 2 | Recent genetic association studies have implicated several candidate susceptibility variants in general populations. Rs1344706, an intronic SNP within ZNF804A, was identified as one of the most compelling risk SNPs in Europeans through genome-wide association studies (GWASs) and replications as well as large-scale meta-analyses. However, in Chinese, the results for rs1344706 are inconsistent, and whether rs1344706 is a real risk SNP for schizophrenia in Chinese is still inconclusive. Here, We conducted a meta-analysis of rs1344706 with schizophrenia in Chinese population by combining all available case-control samples (N=12), including a total of 8,982 cases and 12,342 controls. The results of meta-analysis showed that rs1344706 was not associated with schizophrenia (p=0.10, odds ratio=1.06 for “A” allele), indicating it may not be a risk SNP in Chinese population. Detailed examinations of individual samples revealed potential sampling bias in previous replication studies for schizophrenia in Han Chinese. Collectively, our analysis did not find evidence of significant association between rs1344706 and schizophrenia in Han Chinese, and the effect size of rs1344706 to schizophrenia risk in Han Chinese is smaller than that in Europeans (odds ratio for ‘A’ allele, 1.06 in Chinese vs. 1.12 in Europeans), which unlikely produces significant results in small samples, and calls for large-scale studies of Chinese samples. On the other hand, the failures of replicating rs1344706 in Chinese suggested potential genetic heterogeneity of schizophrenia susceptibility on this locus and also demonstrated the difficulties of replicating genome-wide association findings across different ethnic populations. | 2 |
| **INTRODUCTION** | | |  |
| Rationale | 3 | Rs1344706, an intronic SNP within ZNF804A, was identified as one of the most compelling risk SNPs for schizophrenia in Europeans through genome-wide association studies (GWASs) and replications as well as large-scale meta-analyses. However, in Chinese, the results for rs1344706 are inconsistent, and whether rs1344706 is a real risk SNP for schizophrenia in Chinese is still inconclusive. | 2 |
| Objectives | 4 | We conducted a meta-analysis of rs1344706 with schizophrenia in Chinese population by combining all available case-control samples (N=12), including a total of 8,982 cases and 12,342 controls. | 2 |
| **METHODS** | | |  |
| Protocol and registration | 5 | The review protocol existed in the methods section of the manuscript. | 10 |
| Eligibility criteria | 6 | The included study characteristics have been shown in the Table 1 of the manuscript. | 18 |
| Information sources | 7 | the SZGene database (<http://www.schizophreniaforum.org/>) and the PubMed. | 10 |
| Search | 8 | the SZGene database (<http://www.schizophreniaforum.org/>) and the PubMed (<http://www.ncbi.nlm.nih.gov/>). | 10 |
| Study selection | 9 | Once the article had been collected, their bibliographies were then searched for additional references. Studies published before January 19th, 2013 were considered in the analysis. We read all the relevant papers and see if the studies used Chinese samples. After these preliminary literature search, eighteen non-duplicate studies were identified, including thirteen candidate gene studies using Chinese samples and five GWASs in Han Chinese, but whether they were finally included in the meta-analysis were then determined according to further inclusion criteria as following described. | 10 |
| Data collection process | 10 | Eligible studies in the meta-analysis had to meet all of the following criteria: they had to be: (1) case-control studies, while case-only studies and family-based studies were excluded; (2) with at least 100 cases and 100 controls; (3) case status are defined as having diagnosis of schizophrenia according to the DSM-IV or ICD-10 criterion assessed by established psychiatric interviews, and control subjects had no history of mental disorder, other neurological disorder, alcohol dependence, or drug dependence; (4) studies where the samples have no overlap with the other identified studies; (5) rs1344706 was genotyped and in Hardy-Weinberg equilibrium in healthy controls (p>0.05). | 11 |
| Data items | 11 | For each candidate gene study, the following data was extracted: (1) author(s) and year of publication; (2) methods (study design, sample size, sample collection area, definition of case status, and genotyping method); (3) sample characteristics (gender ratio and mean age); and (4) data for rs1344706 (allele counts). All the required data were available in the published studies or from supplementary information, except for one study without information for mean age and genotyping method.  For the GWASs, we recruited the following data: (1) author(s) and year of publication, (2) methods, and (3) sample characteristics. However, if rs1344706 was not available from the main text or supplementary information of these GWASs, we thus contacted the corresponding authors to request access to the data. The authors of two GWASs provided the data for rs1344706, but the author of another GWAS did not reply to our requests, thus was dropped from further analysis. | 11 |
| Risk of bias in individual studies | 12 | Describe methods used for assessing risk of bias of individual studies (including specification of whether this was done at the study or outcome level), and how this information is to be used in any data synthesis. | 11 |
| Summary measures | 13 | Publication bias was assessed visually using a funnel plot and tested with Egger’s regression test as well as the Begg’s test, which is based on Kendall’s-τ, and p<0.10 was considered to be statistically significant. | 12 |
| Synthesis of results | 14 | To combine the individual studies, we conducted meta-analyses using the Review Manager version 4.2.2. The heterogeneity between individual studies was tested using the Cochran’s (Q) χ2 test, which is a weighted sum of the squares of the deviations of individual odds ratio estimates from the overall estimate. When the odds ratios are homogeneous, Q follows a χ2 distribution with degrees of freedom. If PQ < 0.10, the heterogeneity was considered statistically significant. Inconsistency across studies was quantified with the I2 metric (I2=Q－d.f./Q), which can be interpreted as the percentage of total variation across several studies due to heterogeneity. I2 takes values between 0 and 100%, with higher values denoting a greater degree of heterogeneity (0-25%: no heterogeneity; 25-50%: moderate heterogeneity; 50-75%: large heterogeneity and 75-100%: extreme heterogeneity). In the presence of heterogeneity among individual studies, we used random-effects models to combine the sample and to calculate the odds ratio and the corresponding 95% confidence interval (CI); otherwise, a fixed-effect mode was used. We used a forest plot to graphically present the pooled odds ratios and the 95% CIs. Each study was represented by a square in the plot, and the weight of each study was also shown. P < 0.05 was considered statistically significant. | 12 |

Page 1 of 2

| **Section/topic** | **#** | **Checklist item** | **Reported on page #** |
| --- | --- | --- | --- |
| Risk of bias across studies | 15 | Publication bias was assessed visually using a funnel plot and tested with Egger’s regression test as well as the Begg’s test, which is based on Kendall’s-τ, and p<0.10 was considered to be statistically significant. | 12 |
| Additional analyses | 16 | Sensitivity analysis was conducted to assess the potential influences of any one single study on the pooled odds ratios. Within each meta-analysis, included studies were removed one at a time to check for significant alterations to pooled odds ratios and associated p-values. | 13 |
| **RESULTS** | | |  |
| Study selection | 17 | Using our literature search approaches, a total of nine studies in Chinese population were identified and included in the meta-analysis. Briefly, there are four replication studies for rs1344706 in Chinese having one or two samples, and were included in our meta-analysis. Additionally, there are three previous meta-analyses of rs1344706 in world populations having Chinese samples independently from the four replication studies, and we extracted Shanghai sample from O’Donovan et al. study, Sichuan sample from Steinberg et al. study, and Singapore sample from Li et al. study, and also added these three samples in our meta-analysis. In addition, two Han Chinese GWASs were also included in our meta-analysis. Among these nine studies, a Han Chinese GWAS has three independent samples and a replication study has two independent samples. Therefore, a total of nine studies with twelve independent samples were included in our meta-analysis. | 4-5 |
| Study characteristics | 18 | These information has been included in Table 1 of the manuscript. | 18 |
| Risk of bias within studies | 19 | It should be noted that, there are also some other studies referring ZNF804A and schizophrenia in Han Chinese, however, they were not included in our meta-analysis, either due to small sample size, case-only study, overlap with the samples which have already been included in our meta-analysis, or unavailable from the authors of the studies. | 5 |
| Results of individual studies | 20 | The results have been shown in Figure 3 as a forest plot. | / |
| Synthesis of results | 21 | The results were shown in Table 2 of the manuscript. | 19 |
| Risk of bias across studies | 22 | The STATA statistical software was used to detect the presence of potential publication bias. Begg’s funnel plot (Figure 2) for meta-analysis of rs1344706 seems symmetrical (Begg’s p =0.63), suggesting no evidence of publication bias. Egger’s regression test also shows no publication bias (p=0.14, 95%CI=[-1.16, 7.07]). | 5 |
| Additional analysis | 23 | ‘Leave-one-out’ sensitivity analysis to determine whether the meta-analysis results were driven by any sample data and therefore suggestive of a ‘winner’s curse’ phenomenon revealed that removal of any of the included samples still led to the non-significant (or marginal significant) association of rs1344706 with schizophrenia using the eleven remaining samples (Table S2). Furthermore, we noticed that when the Xi’an sample was removed from the meta-analysis, the estimates of the p-value for the heterogeneity test increased from 0.002 to 0.03, suggesting that the genetic heterogeneity was mainly caused by the Xi’an sample, and when the Xi’an sample was removed from the meta-analysis, the p-value remained non-significant (Z=1.13, p=0.26, odds ratio=1.03, Table S2). | 7 |
| **DISCUSSION** | | |  |
| Summary of evidence | 24 | Through a meta-analysis of all available Han Chinese case-control samples, we did not find evidence for association of rs1344706 with schizophrenia. This is not unexpected given the effect size of rs1344706 to schizophrenia risk in Chinese is smaller than that in Europeans. | 10 |
| Limitations | 25 | The data presented is limited, and we are cautious in the interpretation of our results. In this study, we only tested rs1344706, and the other SNPs in ZNF804A were not studied in most of the analyzed Chinese samples. However, in our previous study, we have performed a relatively systematic analysis of ZNF804A region (up to 111 SNPs) in several Han Chinese samples, which were also included in the present study, i.e., Singapore sample, Yuxi sample, Shanghai and Anhui, Beijing and Shandong, and Guangdong and Guangxi samples, and found that most of the common SNPs in ZNF804A were not associated with schizophrenia in Chinese population. | 10 |
| Conclusions | 26 | Collectively, current data does not support previous speculation that rs1344706 is a risk SNP for schizophrenia in Chinese population, and the failures of replicating rs1344706 in the present sample suggested potential genetic heterogeneity of schizophrenia susceptibility on this locus and also demonstrated the difficulties of replicating genome-wide association findings across different ethnic populations. | 10 |
| **FUNDING** | | |  |
| Funding | 27 | This work was supported by grants from the National 973 project of China (grant numbers, 2011CBA00401) and the National Natural Science Foundation of China (U1202225, 31130051 and 31071101). | / |

*From:*  Moher D, Liberati A, Tetzlaff J, Altman DG, The PRISMA Group (2009). Preferred Reporting Items for Systematic Reviews and Meta-Analyses: The PRISMA Statement. PLoS Med 6(6): e1000097. doi:10.1371/journal.pmed1000097

For more information, visit: **www.prisma-statement.org**.

Page 2 of 2
