# Supplementary material for: Meta-Analysis Indicates That the European GWAS-Identified Risk SNP rs1344706 within ZNF804A Is Not Associated with Schizophrenia in Han Chinese Population
Source: PLoS One. 2013 Jun 12;8(6):e65780. doi: 10.1371/journal.pone.0065780 (PMC3680487; doi:10.1371/journal.pone.0065780)
Supplement: Flow Chart S1 — PRISMA Flow Chart. (DOC) [file pone.0065780.s002.doc]

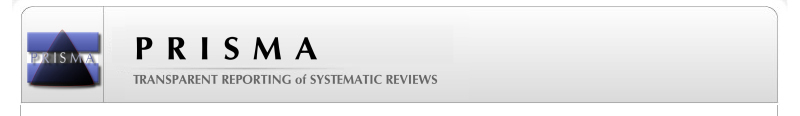
**PRISMA 2009 Flow Diagram**

**Screening**

**Included**

**Eligibility**

**Identification**

Records identified through database searching
(n = 2 )

Additional records identified through other sources
(n = 18 )

Records after duplicates removed
(n = 18 )

Records screened
(n = 18 )

Records excluded
(n = 9 )

Full-text articles assessed for eligibility
(n = 9 )

Full-text articles excluded, with reasons
(n = 0 )

Studies included in qualitative synthesis
(n = 9 )

Studies included in quantitative synthesis (meta-analysis)
(n = 9 )
