# Supplementary material for: Meta-Analysis Indicates That the European GWAS-Identified Risk SNP rs1344706 within ZNF804A Is Not Associated with Schizophrenia in Han Chinese Population
Source: PLoS One. 2013 Jun 12;8(6):e65780. doi: 10.1371/journal.pone.0065780 (PMC3680487; doi:10.1371/journal.pone.0065780)
Supplement: Figure S1 — Power analysis for the studied samples. (DOC) [file pone.0065780.s003.doc]

**Figure S1. Power analysis for the studied samples.**

**
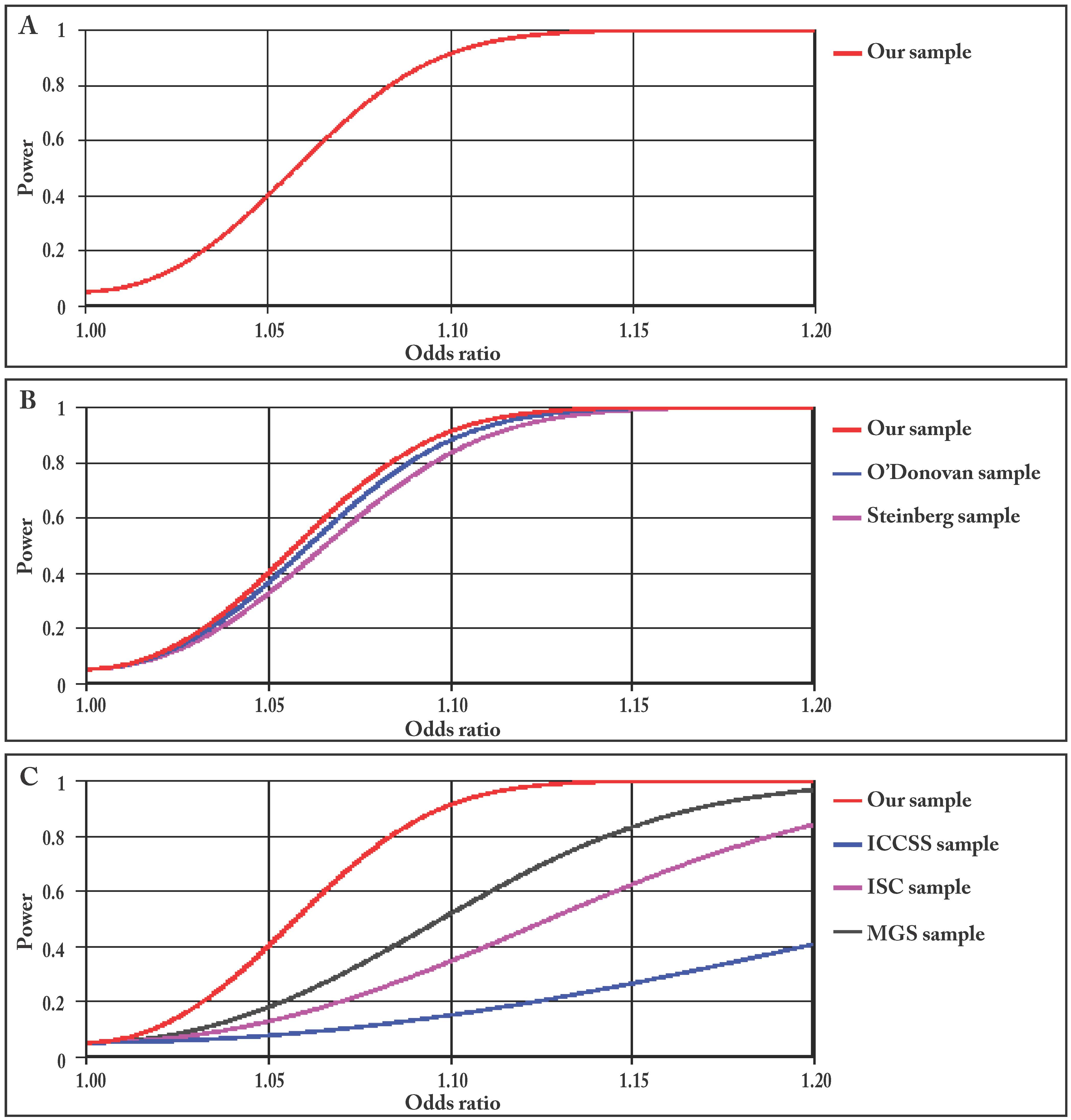
**

**Note:**

(A) Power analysis of our combined sample size;

(B) Comparisons of the power between our sample and the samples used in O’Donovan et al. [15] and Steinberg et al. [17];

(C) Comparisons of the power between our sample and the ICCSS [19], ISC [20] and MGS [21] samples.
