# Supplementary material for: Meta-Analysis Indicates That the European GWAS-Identified Risk SNP rs1344706 within ZNF804A Is Not Associated with Schizophrenia in Han Chinese Population
Source: PLoS One. 2013 Jun 12;8(6):e65780. doi: 10.1371/journal.pone.0065780 (PMC3680487; doi:10.1371/journal.pone.0065780)
Supplement: Table S1 — Studies referring ZNF804A and schizophrenia in Han Chinese, but not meet the inclusion criteria for the meta-analysis. (DOC) [file pone.0065780.s004.doc]

**Table S1**. Studies referring ZNF804A and schizophrenia in Han Chinese, but not meet the inclusion criteria for the meta-analysis

| **Author, year** | **Study types*** | **Title of the excluded studies** | **Reason for exclusion** |
| --- | --- | --- | --- |
| Kuswanto, 2012 [1] | CGS | Genome-wide supported psychosis risk variant in ZNF804A gene and impact on cortico-limbic WM integrity in schizophrenia | Sample size is too small |
| Ma, 2011 [2] | GWAS | A genome-wide association study for quantitative traits in schizophrenia in China | Sample size is too small |
| Wei, 2012 [3] | CGS | Association of the ZNF804A gene polymorphism rs1344706 with white matter density changes in Chinese schizophrenia | Sample size is too small |
| Wei, 2012 [4] | CGS | No association of ZNF804A rs1344706 with white matter integrity in schizophrenia: A tract-based spatial statistics study | Sample size is too small |
| Xiang, 2012 [5] | GWAS | Genome-wide association study with memory measures as a quantitative trait locus for schizophrenia | Sample size is too small |
| Yue, 2011 [6] | GWAS | Genome-wide association study identifies a susceptibility locus for schizophrenia in Han Chinese at 11p11.2 | unavailable from author |
| Zhang, 2011 [7] | CGS | Is the conserved mammalian region of ZNF804A locus associated with schizophrenia? A population-based genetics analysis | Overlap with Zhang, 2010 [8] |
| Zhang, 2012 [9] | CGS | Association analysis of ZNF804A (zinc finger protein 804A) rs1344706 with therapeutic response to atypical antipsychotics in first-episode Chinese patients with schizophrenia | Case-only study |
| Zhang, 2012 [10] | CGS | Further evidence for the association of genetic variants of ZNF804A with schizophrenia and a meta-analysis for genome-wide significance variant rs1344706 | Overlap with Zhang, 2010 [8] |

*CGS, candidate gene study; GWAS, genome-wide association study

**References**

1. Kuswanto CN, Woon PS, Zheng XB, Qiu A, Sitoh YY, et al. (2012) Genome-wide supported psychosis risk variant in ZNF804A gene and impact on cortico-limbic WM integrity in schizophrenia. Am J Med Genet B Neuropsychiatr Genet 159B: 255-262.

2. Ma X, Deng W, Liu X, Li M, Chen Z, et al. (2011) A genome-wide association study for quantitative traits in schizophrenia in China. Genes Brain Behav 10: 734-739.

3. Wei Q, Kang Z, Diao F, Shan B, Li L, et al. (2011) Association of the ZNF804A gene polymorphism rs1344706 with white matter density changes in Chinese schizophrenia. Prog Neuropsychopharmacol Biol Psychiatry 36: 122-127.

4. Wei Q, Kang Z, Diao F, Guidon A, Wu X, et al. (2012) No association of ZNF804A rs1344706 with white matter integrity in schizophrenia: A tract-based spatial statistics study. Neurosci Lett 10.1016/j.neulet.2012.10.062.

5. Xiang B, Wu J, Ma X, Wang Y, Deng W, et al. (2012) Genome-wide association study with memory measures as a quantitative trait locus for schizophrenia. Chinese journal of medical genetics 29: 255.

6. Yue WH, Wang HF, Sun LD, Tang FL, Liu ZH, et al. (2011) Genome-wide association study identifies a susceptibility locus for schizophrenia in Han Chinese at 11p11.2. Nat Genet 43: 1228-1231.

7. Zhang R, Valenzuela RK, Lu S, Meng L, Guo T, et al. (2011) Is the conserved mammalian region of ZNF804A locus associated with schizophrenia? A population-based genetics analysis. Schizophr Res 133: 159-164.

8. Zhang R, Lu SM, Qiu C, Liu XG, Gao CG, et al. (2011) Population-based and family-based association studies of ZNF804A locus and schizophrenia. Mol Psychiatry 16: 360-361.

9. Zhang J, Wu X, Diao F, Gan Z, Zhong Z, et al. (2012) Association analysis of ZNF804A (zinc finger protein 804A) rs1344706 with therapeutic response to atypical antipsychotics in first-episode Chinese patients with schizophrenia. Compr Psychiatry 53: 1044-1048.

10. Zhang R, Yan JD, Valenzuela RK, Lu SM, Du XY, et al. (2012) Further evidence for the association of genetic variants of ZNF804A with schizophrenia and a meta-analysis for genome-wide significance variant rs1344706. Schizophr Res 141: 40-47.

11. Chen M, Xu Z, Zhai J, Bao X, Zhang Q, et al. (2012) Evidence of IQ-modulated association between ZNF804A gene polymorphism and cognitive function in schizophrenia patients. Neuropsychopharmacology 37: 1572-1578.

12. Li M, Luo XJ, Xiao X, Shi L, Liu XY, et al. (2011) Allelic differences between Han Chinese and Europeans for functional variants in ZNF804A and their association with schizophrenia. Am J Psychiatry 168: 1318-1325.

13. Li M, Shi CJ, Shi YY, Luo XJ, Zheng XB, et al. (2012) ZNF804A and schizophrenia susceptibility in Asian populations. Am J Med Genet B Neuropsychiatr Genet 159B: 794-802.

14. Liou YJ, Wang HH, Lee MT, Wang SC, Chiang HL, et al. (2012) Genome-wide association study of treatment refractory schizophrenia in Han Chinese. PLoS One 7: e33598.

15. O'Donovan MC, Craddock N, Norton N, Williams H, Peirce T, et al. (2008) Identification of loci associated with schizophrenia by genome-wide association and follow-up. Nat Genet 40: 1053-1055.

16. Shi Y, Li Z, Xu Q, Wang T, Li T, et al. (2011) Common variants on 8p12 and 1q24.2 confer risk of schizophrenia. Nat Genet 43: 1224-1227.

17. Steinberg S, Mors O, Borglum AD, Gustafsson O, Werge T, et al. (2011) Expanding the range of ZNF804A variants conferring risk of psychosis. Mol Psychiatry 16: 59-66.

18. Xiao B, Li W, Zhang H, Lv L, Song X, et al. (2011) Association of ZNF804A polymorphisms with schizophrenia and antipsychotic drug efficacy in a Chinese Han population. Psychiatry Res 190: 379-381.

19. Riley B, Thiselton D, Maher BS, Bigdeli T, Wormley B, et al. (2010) Replication of association between schizophrenia and ZNF804A in the Irish Case-Control Study of Schizophrenia sample. Mol Psychiatry 15: 29-37.

20. Purcell SM, Wray NR, Stone JL, Visscher PM, O'Donovan MC, et al. (2009) Common polygenic variation contributes to risk of schizophrenia and bipolar disorder. Nature 460: 748-752.

21. Shi J, Levinson DF, Duan J, Sanders AR, Zheng Y, et al. (2009) Common variants on chromosome 6p22.1 are associated with schizophrenia. Nature 460: 753-757.
